# Carpets with visual cues can improve gait in Parkinson’s disease patients: may be independent of executive function

**DOI:** 10.1186/s40001-023-01472-1

**Published:** 2023-11-16

**Authors:** Ze-Di Hu, Shi-Guo Zhu, Jie-Fan Huang, Jin-Yu Chen, Shi-Shi Huang, Rong-Pei Liu, Zhu-Ling Chen, Lu-Lu Ma, Xiong Zhang, Jian-Yong Wang

**Affiliations:** Department of Neurology, Institute of Geriatric Neurology, The Second Affiliated Hospital and Yuying Children’s Hospital, Wenzhou Medical University, Wenzhou, Zhejiang China

**Keywords:** Parkinson’s disease, Gait impairment, Visual cues, Executive function, Compensation strategy

## Abstract

**Background:**

Gait impairment is common in Parkinson’s disease (PD) patients, which greatly reduces their quality of life. Executive dysfunction is associated with gait impairment. Compensatory strategies, including visual cues, have been shown to be effective in improving PD gait. In this study, we aimed to understand whether carpets with visual cues could improve PD gait, and how the improvement varies across patients with different executive function state.

**Methods:**

We designed carpets with chessboard and stripe cues. A total of 65 Chinese PD patients were recruited. Movement Disorder Society Unified Parkinson’s Disease Rating Scale, L-dopa equivalent daily dosage, Hoehn & Yahr stage, Frontal Assessment Battery, Mini Mental State Examination Scale, Hamilton Anxiety Scale, and Hamilton Depression Scale were evaluated. Gait parameters including stride length, gait speed and fall risk were recorded by a wearable electronic device.

**Results:**

The stride length and gait speed were significantly improved and the fall risk was significantly mitigated when PD patients walked on carpets with chessboard and stripe patterns. Further analysis showed the amelioration of gait parameters was independent of executive dysfunction.

**Conclusions:**

Our study demonstrates that carpets with visual cues can improve the gait of PD patients even in those with mild executive dysfunction.

## Background

Parkinson’s disease (PD) is a common neurodegenerative disease, whose prevalence rises rapidly with age [1]. The cardinal motor manifestations of PD include bradykinesia, rigidity, rest tremor, and gait impairment [2]. Gait disturbance runs through the course of PD and greatly affects their quality of life [3].

The characteristics of gait impairment in PD patients vary widely, including decreased speed, reduced stride length, reduced arm swing amplitude, increased interlimb asymmetry, larger gait variability, impaired complex locomotor tasks, reduced axial rotation, stooped posture, impaired motor automaticity, freezing of gait (FOG), and increased risk of falling [3–5]. It has been believed that gait impairment in PD patients is not only related to deficit of motor activity, but also closely related to higher-level cognitive impairments such as executive dysfunction [6, 7]. Accurate assessment and treatment of gait impairment in PD patients is very important.

Several observational and quantitative scales such as Movement Disorder Society Unified Parkinson’s Disease Rating Scale (MDS-UPDRS) [8], Freezing of Gait Questionnaire [9], Timed Up-and-Go [10], and 6-min walk test [11] have been widely used to assess PD gait. However, these methods obtain relatively simple metrics and are susceptible to tester bias [3, 12]. Sensor-based wearable electronic devices enable gait measures more detailed and can be performed in home and community settings.

Similar to bradykinesia, tremor, and rigidity, gait impairment in PD can be clearly relieved by dopaminergic drugs, especially in the early stage. However, these drugs are not always effective and might lead to sedation, orthostatic hypotension, behavioral and psychiatric symptoms [13, 14]. Motor fluctuation and dyskinesia associated with long-term use of levodopa can even lead to worsening of gait [15]. Therefore, non-pharmacological interventions to treat PD gait are receiving increasing attention. Numerous studies have shown that various aspects of gait in PD patients can be improved by different rhythmical visual and auditory cues [16–20]. A previous study showed that public space floors with visual cues can improve gait in PD patients [21].

In this study, we designed carpets with visual cues, and aimed to explore whether they could improve gait in PD patients using wearable electronic device. We also want to test if executive dysfunction affects the gait improvement.

## Methods

### Patients

A total of 65 PD patients of Han Chinese ethnicity were recruited from Department of Neurology, the Second Affiliated Hospital and Yuying Children’s Hospital from February 2021 to March 2023. All patients were diagnosed by two movement disorder neurologists according to the movement disorder society clinical diagnostic criteria for PD [22]. Most of them were de novo PD patients. Excluded participants were those with severe cognitive impairment, uncorrectable visual disorders, neuromusculoskeletal disorders that affect gait, and severe gait disorder that prevents independent walking. Since the automated device cannot distinguish FOG, we also excluded patients with FOG based on Freezing of Gait Questionnaire.

### Clinical evaluations

Clinical information including age, disease duration, MDS-UPDRS, L-dopa equivalent daily dosage (LEDD), Hoehn & Yahr stage, Frontal Assessment Battery (FAB), Mini Mental State Examination Scale (MMSE), Hamilton Anxiety Scale (HAMA), and Hamilton Depression Scale (HAMD) were assessed by face-to-face interview and physical examinations in the off medication state, which is defined as at least 12 h after stopping antiparkinsonian medication [23]. All the patients were evaluated with FAB [24] and they were divided into PD with executive dysfunction group (FAB score < 13) and PD without executive dysfunction group (FAB score ≥ 13) based on the FAB score, as previously described [25].

### Gait analysis

**Fig. 1:**
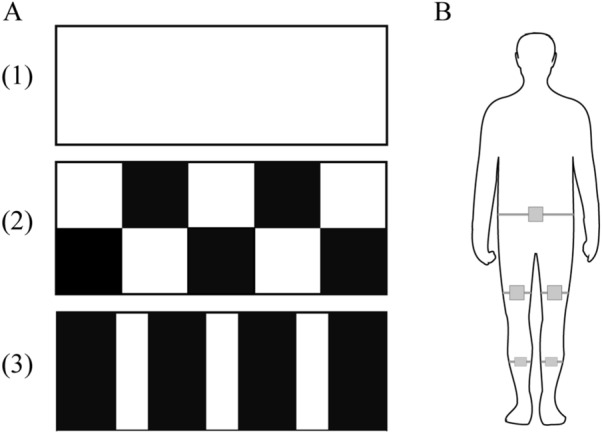
Three patterns of carpets and position of wearable sensor modules. **A** (1) No pattern; (2) Chessboard pattern; (3) Stripe pattern. **B** Participants wore five sensor modules on the waist, both thighs, and calves

The 5-m-long and 1-m-wide carpet is designed into 3 patterns (Fig. 1A): (1) No pattern (blank carpet); (2) Chessboard pattern (50 × 50 cm black-white chessboard); (3) Stripe pattern (50 cm black and 25 cm white spaced stripes). To better reflect daily life, patients were asked to walk three times at a natural speed on each pattern of carpet in their habitual on medication state, when they felt as comfortable as usual. Gait performance of the patients was evaluated by a wearable electronic device (Dalian Qianhan Technology Co., Limited, China), which was widely used in PD gait evaluation [26, 27]. Participants were asked to wear five sensor transmission modules on the waist, both thighs, and calves (Fig. 1B). Parameters including stride length, gait speed were calculated according to the acceleration data automatically. Fall risk was calculated by the system according to the gait parameters as previously described [28, 29].

### Statistical analysis

The data were analyzed using IBM SPSS Statistics 19.0 for windows. The normal distribution of the data was assessed by Kolmogorov–Smirnov test. Variables were described as mean ± standard deviation (SD) when the data are normally distributed. Otherwise, they were described as the median and interquartile range (IR). Difference in gender between PD with executive dysfunction and PD without executive dysfunction groups was assessed by Chi square test. Differences in age, MDS-UPDRS total score, and MDS-UPDRS III score were analyzed by unpaired two-tailed t-test. Differences in disease duration, LEDD, Hoehn & Yahr stage, FAB score, MMSE score, HAMA score, and HAMD score were analyzed by Mann–Whitney U test. Differences in stride length and gait speed when patients walked on three patterned carpets were analyzed by One-way repeated measures ANOVA. Two-way repeated measures ANOVA test was used to analyze the interaction between executive function and visual cues on gait parameters. Difference in fall risk when patients walked on three patterned carpets was analyzed by Friedman test. A two-tailed *P* < 0.05 was considered statistically significant.

## Results

### Demographic and clinical characteristics of PD patients

According to the FAB score, there were 24 cases in PD with executive dysfunction group and 41 cases in PD without executive dysfunction group. The two groups were comparable in gender, age, disease duration, MDS-UPDRS total score, MDS-UPDRS III score, LEDD, Hoehn & Yahr stage, HAMA score and HAMD score (*P* > 0.05; Table 1). In contrast, FAB score and MMSE score were significantly lower in PD with executive dysfunction group (*P* < 0.001 and *P* = 0.001 respectively; Table 1).

**Table 1:** Demographic and clinical characteristics of PD patients

	PD with executive dysfunction	PD without executive dysfunction	P^*^
Subject, *n* (%)	24 (36.9)	41 (63.1)	–
Gender, F/M	9/15	15/26	0.941^a^
Age, mean ± SD	67.7 ± 10.0	63.4 ± 10.1	0.105^b^
Duration, years (IR)	3.0 (2.0–5.75)	3.0 (1.5–5.0)	0.568^c^
MDS-UPDRS total, mean ± SD	62.7 ± 23.4	54.7 ± 28.4	0.248^b^
MDS-UPDRS III, mean ± SD	36.3 ± 15.3	32.6 ± 17.8	0.403^b^
LEDD, mg (IR)	0.0 (0.0–300.0)	0.0 (0.0–300.0)	0.769^c^
Hoehn & Yahr, (IR)	2.0 (1.625–2.5)	2.0 (1.5–2.5)	0.480^c^
FAB, (IR)	10.0 (9.0–11.0)	15.0 (13.0–16.0)	< 0.001^c^
MMSE, (IR)	26.0 (24.0–27.0)	28.0 (27.0–29.0)	0.001^c^
HAMA, (IR)	8.0 (5.0–15.75)	8.0 (5.0–13.5)	0.989^c^
HAMD, (IR)	6.5 (4.25–11.0)	7.0 (2.5–12.0)	0.865^c^

F: female; HAMA: Hamilton Anxiety Scale; HAMD: Hamilton Depression Scale; IR: interquartile range; LEDD: L-dopa equivalent daily dosage; M: male; MDS-UPDRS: Movement Disorder Society-Unified Parkinson’s disease Rating Scale; MMSE: Mini Mental State Examination Scale; PD: Parkinson’s disease; SD: standard deviation

^*^Compared between PD patients with and without executive dysfunction groups

^a^Analyzed by Chi square test

^b^Analyzed by unpaired two-tailed *t*-test

^c^Analyzed by Mann–Whitney U test

### Carpets with chessboard and striped patterns improve PD gait

**Fig. 2:**
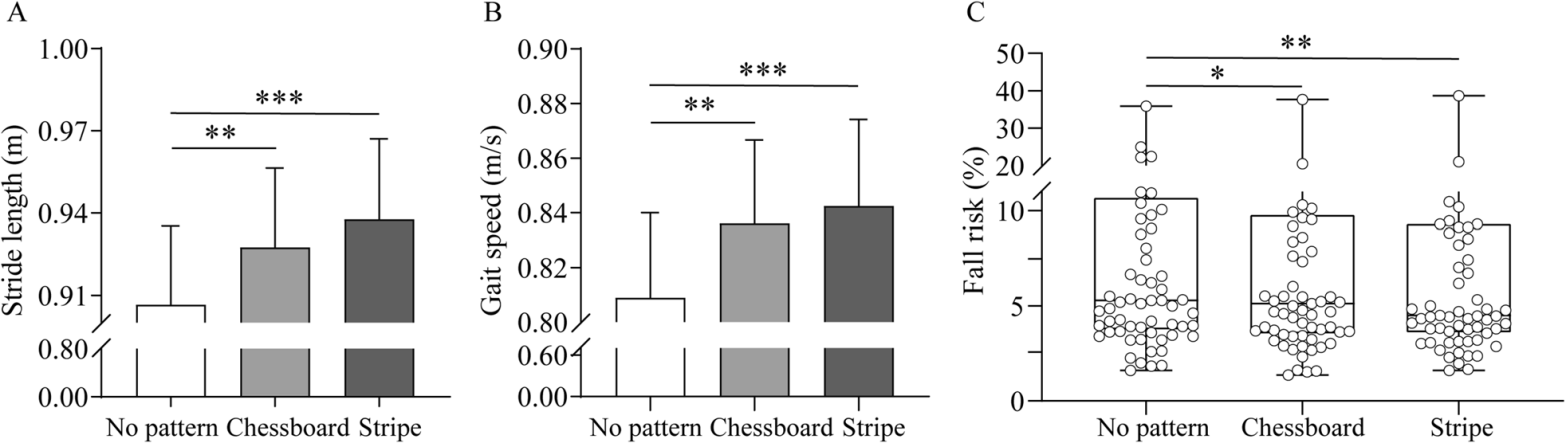
Comparison of gait parameters among three patterned carpets. **A** Stride length. **B** Gait speed. **C** Fall risk. *n* = 65. Values are expressed as means ± SE for stride length and gait speed. Data of fall risk is expressed as median and interquartile range. *P < 0.05; **P < 0.01; ***P < 0.001

Stride length, gait speed and fall risk of the 65 PD patients were recorded and analyzed when they walked on carpets with three patterns. The stride length was 0.905 ± 0.029 m when they walked on carpet with no pattern, which was significantly shorter than the stride length when they walked on carpets with chessboard and stripe patterns (0.927 ± 0.029 m, *P* = 0.001 and 0.938 ± 0.029 m, *P* < 0.001 respectively; Fig. 2A). Gait speed was also significantly faster when they walked on chessboard and stripe patterned carpets than on carpet with no pattern (0.836 ± 0.030 m/s vs. 0.809 ± 0.031 m/s, *P* = 0.008 and 0.842 ± 0.032 m/s vs. 0.809 ± 0.031 m/s, *P* < 0.001 respectively; Fig. 2B). The fall risk on carpets with no pattern, chessboard and stripe patterns was 5.300% (interquartile range, 3.767–10.667%), 5.133% (interquartile range, 3.567–9.782%), and 4.500% (interquartile range, 3.619–9.300%) respectively. Both chessboard and stripe patterns were statistically different from no pattern (*P* = 0.037 and *P* = 0.001 respectively; Fig. 2C). However, no significant differences were found between the gait parameters on the 2 patterned carpets (*P* > 0.05).

### Analysis of executive function on gait improvement

**Fig. 3:**
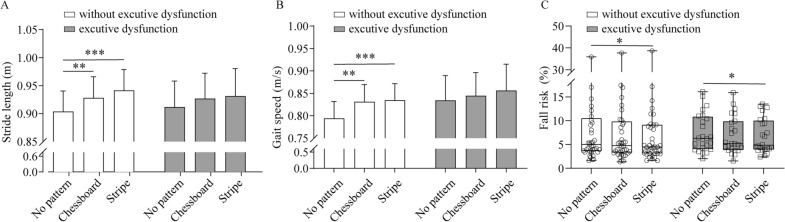
Change of gait parameters induced by the designed carpets in PD patients with and without executive dysfunction. **A** Stride length. **B** Gait speed. **C** Fall risk. *n* = 24 for PD patients with executive dysfunction; *n* = 41 for PD patients without executive dysfunction. Values are expressed as means ± SE for stride length and gait speed. Data of fall risk is expressed as median and interquartile range. *P < 0.05; **P < 0.01; ***P < 0.001

In the further analysis, patients were divided into PD with executive dysfunction group and PD without executive dysfunction group according to their FAB scores. PD patients without executive dysfunction showed significant stride length improvement on chessboard and stripe patterned carpets (*P* = 0.003 and *P* < 0.001 respectively; Fig. 3A). Similar results were found in gait speed (*P* = 0.004 and *P* < 0.001 respectively; Fig. 3B). Significant amelioration of fall risk was found in both groups of PD patients with and without executive dysfunction when they walked on carpet with stripe pattern (*P* = 0.035 and *P* = 0.020 respectively; Fig. 3C). To further explore the effect of executive function on stride length and gait speed improvements, a two-way repeated measures ANOVA was performed. As presented in Table 2, both stride length and gait speed were significantly benefited from visual cues (*P* < 0.001 and *P* < 0.001 respectively). However, there was no significant interaction between visual cues and executive dysfunction (*P* = 0.359 and *P* = 0.270 respectively; Table 2), or significant main effect of executive dysfunction (*P* = 0.982 and *P* = 0.694 respectively; Table 2).

**Table 2:** Results of two-way repeated measures ANOVA

Variables	Stride length	Gait speed
VC, *P*	< 0.001	< 0.001
ED, *P*	0.982	0.694
VC × ED, *P*	0.359	0.270

ED: executive dysfunction; VC: visual cues

## Discussion

Gait impairment is very common and heterogeneous in PD patients. In this study, we designed two kinds of carpets with visual cues (chessboard pattern and stripe pattern), and found that carpets with the two patterns are effective in improving gait in PD patients without FOG. Further analysis suggested that this improvement may be independent of executive function.

Gait impairment is a major concern for PD patients, causing falls and recurrent falls in 68.3% and 39% of the patients [30–32]. Treatment for PD gait impairment is diverse. Although their therapeutic targets are different, levodopa, dopamine agonists, inhibitors of amine oxidase B, and acetylcholinesterase inhibitors have all been reported to be effective in improving PD gait [13, 33–36]. However, the side effects of long-term use and the uncertainty of efficacy are their limitations [13]. Non-pharmacological interventions make up for these deficiencies and bring additional benefits. Compensation strategies developed spontaneously or trained by doctors can alleviate PD gait impairment by bypassing the basal ganglia pathway, among which, external cues are known to be the most widely applied strategy [37, 38]. External cues refer to a series of rhythmically visual, auditory, or proprioceptive stimuli that provide a gait reference. To better apply visual cue strategies to home and community settings, we designed carpets with chessboard and striped cues in the current study. Based on wearable device recordings and subsequent analysis, our results showed that the two patterned carpets can improve stride length, gait speed and fall risk in PD patients. A previous study designed a chessboard floor and tested it in 32 PD patients with FOG. The results showed that it can improve several parameters of gait [21]. Our study, with a larger sample size, showed that even PD patients without FOG would benefit from carpets with visual cues.

The underlying mechanism of gait impairments in PD patients is not clear. Relevant studies are mainly carried out in cross-sectional studies, focusing on brain functional MRI, risk factors, and neurotransmitters. In addition to the nigrostriatal dopamine system, non-motor symptoms, non-dopaminergic systems, and frontal-striatal connections have also been shown to be associated with gait impairment in PD [39–41]. Executive dysfunction plays an important role in gait impairment [7]. A previous study showed that both mild and marked executive dysfunctions are independent risk factors for falls in PD patients [25]. What’s more, rivastigmine and donepezil, which are used to improve executive function, have been shown to be effective in improving PD gait [35, 36].

Highlighted should be that we firstly evaluated the gait improvement by visual cues in PD patients with or without executive dysfunction respectively. The results showed that the benefits in stride length and gait speed are significant in those without executive dysfunction. However, further analysis showed that this improvement was not significantly different between the patients with and without executive dysfunction. It may be due to the fact that executive dysfunction is not extremely severe in our cohort, as we have excluded those who could not follow the instructions when recruiting patients. Our results suggest that the gait-improving effect of visual cues is also valuable in PD patients with mild executive dysfunction.

It is worth noting that a short walk (in the absence of habituation) on a patterned carpet may raise attention, and further boost the gait performance. The habits of walking on such patterns may induce habituation, lower attention and eventually lose the benefit observed on gait in such a research setting. Therefore, whether such patterned carpets can permanently improve PD gait needs further study. Meanwhile, we have to acknowledge certain limitations. The turns, narrow spaces and obstacles that exist in real home and community settings are not reflected in our research. In addition, our study has a relatively small sample size and lacks longitudinal observation, the presence of which would otherwise enhance the reliability of the conclusions.

## Conclusions

In summary, the current study provides further evidence that carpets with visual cues can improve the gait of PD patients even in those with mild executive dysfunction. Further study in larger population is needed to confirm our findings.

## Data Availability

The original contributions presented in the study are included in the article, further inquiries can be directed to the corresponding authors.

## Abbreviations

ED: Executive dysfunction
F: Female
FOG: Freezing of gait
HAMA: Hamilton Anxiety Scale
HAMD: Hamilton Depression Scale
IR: Interquartile range
LEDD: L-dopa equivalent daily dosage
M: Male
MDS-UPDRS: Movement Disorder Society-Unified Parkinson’s disease Rating Scale
MMSE: Mini Mental State Examination Scale
PD: Parkinson’s disease
SD: Standard deviation
VC: Visual cues
